# 顶空-气相色谱法测定伊班膦酸钠中多种残留溶剂

**DOI:** 10.3724/SP.J.1123.2024.01023

**Published:** 2024-05-08

**Authors:** Ran ZHOU, Fei WANG, Jiarui LIN, Meng JIA, Yan WANG

**Affiliations:** 1.石家庄学院化工学院, 河北 石家庄 050035; 1. College of Chemical Engineering, Shijiazhuang University, Shijiazhuang 050035, China; 2.石家庄学院科研处, 河北 石家庄 050035; 2. Research Department, Shijiazhuang University, Shijiazhuang 050035, China

**Keywords:** 顶空-气相色谱法, 残留溶剂, 1-戊醇, 伊班膦酸钠, headspace-gas chromatography (HS-GC), residual solvents, 1-pentanol, ibandronate sodium

## Abstract

采用顶空-气相色谱建立了同时测定伊班膦酸钠原料药中5种残留溶剂含量的方法。以Agilent DB-624毛细管色谱柱(30 m×0.32 mm×1.8 μm)为分离柱,考察了顶空平衡温度、平衡时间等对残留溶剂测定的影响。实验结果表明,在顶空平衡温度为80 ℃、平衡时间为20 min的条件下,甲醇、丙酮、苯、甲苯、1-戊醇的质量浓度与峰面积之间存在良好的线性关系,相关系数*r*值均大于0.990。各溶剂的检出限依次为2.88、0.011、0.90、0.24、0.024 ng/mL,定量限依次为11.5、0.043、3.6、0.96、0.096 ng/mL。各溶剂的回收率为86.3%~101.9%,相对标准偏差(*n*=3)均小于2.49%。该方法操作简便、准确可靠,适用于伊班膦酸钠原料药中5种残留溶剂的同时快速测定。

## Abstract (en)

Ibandronate sodium, a third-generation diphosphate drug used worldwide to treat osteoporosis, has the advantages of convenient use, low toxicity, and significant therapeutic effects. However, the residual organic solvents in the synthesis process of sodium ibandronate not only have a negative impact on the efficacy of the drug, but also lead to a decrease in drug stability. Moreover, if the residual amounts of these solvents exceed safety standards, they may pose serious threats to human health. This study successfully established a convenient and efficient method based on headspace-gas chromatography (HS-GC) for the simultaneous determination of five residual solvents (methanol, acetone, benzene, toluene, 1-pentanol) in the raw materials of ibandronate sodium. The results indicated that satisfactory analytical performance can be achieved by using DB-624 capillary column (30 m×0.32 mm×1.8 μm) and a flame ionization detector in conjunction with headspace autosampling and a temperature program. The specific operating conditions included an initial temperature of 40 ℃, with a hold of 2 min, followed by a temperature ramp first to 200 ℃ at a rate of 5 ℃/min and then to 240 ℃ at a rate of 20 ℃/min, with a hold of 5 min. Nitrogen with a flow rate of 1 mL/min and split ratio of 14∶1 was used as the carrier gas. The headspace vial temperature was maintained at 80 ℃, and the sample equilibration time was 20 min. Under the established analytical conditions, good linear relationships were obtained between the mass concentrations of methanol (72-216 μg/mL), acetone (120-360 μg/mL), benzene (0.048-0.144 μg/mL), toluene (21.36-64.08 μg/mL), and 1-pentanol (120-360 μg/mL) and their corresponding peak areas, with correlation coefficients (*r*) greater than 0.990. The limits of detection for these solvents were 2.88, 0.011, 0.90, 0.24, and 0.024 ng/mL, respectively, with limits of quantification of 11.5, 0.043, 3.6, 0.96, and 0.096 ng/mL, respectively. Furthermore, the recoveries of these solvents ranged from 86.3% to 101.9%, with relative standard deviations (RSDs, *n*=3) of less than 2.49%. The proposed method is simple, accurate, reliable, and suitable for the rapid and simultaneous determination of five residual solvents in the raw materials of ibandronate sodium. This study has important practical significance in improving drug safety and ensuring public health.

19世纪中期,双膦酸盐,即二磷酸的第一个类似物,被首次开发并在工业中被广泛用作金属螯合剂和水软化剂。然而,直到20世纪中期,人们才开始认识到它们对骨组织和破骨细胞的生物学作用,进一步推动了新型结构的开发^[1,2]^。这些新结构化合物主要包括唑来膦酸盐、利塞膦酸盐、阿仑膦酸盐、帕米膦酸盐、因卡膦酸盐和伊班膦酸盐^[3⇓-5]^。

伊班膦酸钠作为一种合成的含氮双膦酸药物,属于第三代二膦酸盐类药物,具有低毒性和出色的骨重吸收抑制效果。它在肿瘤引起的高钙血症治疗中表现出色,同时也是治疗骨质疏松症的主要药物之一。与其他同类药物相比,伊班膦酸钠具有低毒性和显著的治疗效果,因此在国际上应用广泛^[6⇓-8]^。文献中有少数关于伊班膦酸钠的分析报道,仅限于对其杂质和含量的测定研究^[9,10]^。然而,在伊班膦酸钠原料药的制备过程中,国内外公开了多条合成工艺路线,多种有机溶剂的使用成为不容忽视的问题。这些溶剂包括甲醇、丙酮、甲苯和1-戊醇等,在合成过程中难以完全去除,可能对药物的疗效和稳定性造成负面影响。更为严重的是,若这些残留溶剂的含量超出安全标准,则可能对人体健康造成危害^[11⇓-13]^。此外,甲醇、丙酮和甲苯的使用过程中可能会产生苯,而苯的毒性较大,因此需要特别关注其含量。

根据《中国药典》2020年版及《化学药物残留溶剂研究技术指导原则》对各有机溶剂限度的明确规定,苯被归类为第一类有机溶剂,应尽量避免使用,其限度设定为0.0002%;甲醇和甲苯属于第二类有机溶剂,应限制使用,限度分别为0.3%和0.089%;丙酮和1-戊醇则属于第三类有机溶剂,由于其低毒性,是推荐使用的溶剂,其限度均为0.5%^[14]^。虽然已有研究尝试通过毛细管气相色谱法(GC)和程序升温GC来测定伊班膦酸钠中部分有机溶剂的残留量,如李晓昕等^[15]^以正丙醇为内标物,建立了毛细管GC测定伊班膦酸钠中的丙酮、甲醇、乙醇、氯苯4种有机溶剂残留量的方法;谭芳等^[16]^应用程序升温GC同时测定了伊班膦酸钠原料中甲醇、乙醇、丙酮、甲苯4种有机残留溶剂的含量。但这些研究仅进行了简单的方法学描述,缺乏准确的方法耐用性数据。此外,《中国药典》中尚未收录伊班膦酸钠原料药及其制剂的质量标准,说明在这一领域还存在着明显的研究和标准化空白。

顶空-气相色谱法(HS-GC)是一种用于残留溶剂检测的有效方法,具有出色的分离性能、高灵敏度和实际应用性。我们结合伊班膦酸钠原料药的制备工艺,根据《人用药品注册技术国际协调会Q3C指导原则(ICHQ3C)》^[17]^要求和《化学药物残留溶剂研究的技术指导原则》,建立了同时快速分析伊班膦酸钠原料药中5种有机溶剂(甲醇、丙酮、苯、甲苯、1-戊醇)残留的HS-GC方法。

## 1 实验部分

### 1.1 仪器、材料与试剂

Agilent 7890B-7697A型顶空-气相色谱仪,配氢火焰离子化检测器(FID)(美国Agilent公司); KQ-100B型超声波清洗仪(昆山市超声仪器有限公司); XS-105du型分析天平(梅特勒-托利多公司);顶空瓶(体积22 mL,美国Agilent公司)。

伊班膦酸钠原料药(纯度大于99.0%,河北医科大学制药厂);二甲基乙酰胺(DMA)、甲醇、丙酮、苯、甲苯、1-戊醇均为分析纯(上海麦克林化学试剂厂);水为超纯水。

### 1.2 溶液的制备

#### 1.2.1 空白溶液

精密量取DMA 1.0 mL,置于100 mL量瓶中,加入20 g/L的氯化钠溶液并稀释至刻度,摇匀。再精密量取上述溶液5.0 mL,置于顶空瓶中,作为空白溶液。

#### 1.2.2 混合标准溶液

精密量取5种溶剂适量,置于同一量瓶中,用DMA稀释成含甲醇18 mg/mL、丙酮30 mg/mL、苯0.012 mg/mL、甲苯5.34 mg/mL、1-戊醇30 mg/mL的混合对照品储备液。分别精密量取对照品储备液0.4、0.5、0.6、0.8、1.0、1.2 mL,置于100 mL量瓶中,加入20 g/L的氯化钠溶液并稀释至刻度,摇匀,得到6种不同质量浓度的系列混合标准溶液。

#### 1.2.3 供试品溶液

取伊班膦酸钠原料药约300 mg,精密称定,置于顶空瓶中,精密加入混合对照品溶液5.0 mL使其溶解,密封,摇匀,作为供试品溶液。

### 1.3 分析条件

#### 1.3.1 气相色谱条件

DB-624石英毛细管色谱柱(30 m×0.32 mm×1.8 μm, 6%氰丙基苯基-94%二甲基硅氧烷,美国Agilent公司)。采用程序升温:初始温度40 ℃,保持2 min,以5 ℃/min速率升温至200 ℃,再以20 ℃/min速率升温至240 ℃,保持5 min;以氮气为载气,流速1.0 mL/min,分流比14∶1;进样口温度200 ℃, FID温度260 ℃。

#### 1.3.2 顶空条件

箱体温度:80 ℃,定量环温度:90 ℃,传输线温度:100 ℃;样品平衡时间:20 min, GC循环时间:55 min。

## 2 结果与讨论

### 2.1 顶空条件的优化

#### 2.1.1 顶空平衡温度

气液分配常数会随温度的变化而改变。在一定的温度范围内,随着温度的升高,目标物在气相中的平衡浓度也会增加。然而,平衡温度过高则会引起杂质峰的干扰,并可能破坏气液平衡的稳定性,导致分析时间延长^[18]^。分别取供试品溶液在60、70、80、90、100、110、120 ℃ 7个温度条件下进行GC测定,考察顶空平衡温度的变化对各残留溶剂峰面积的影响,结果见图1。

**图1:**
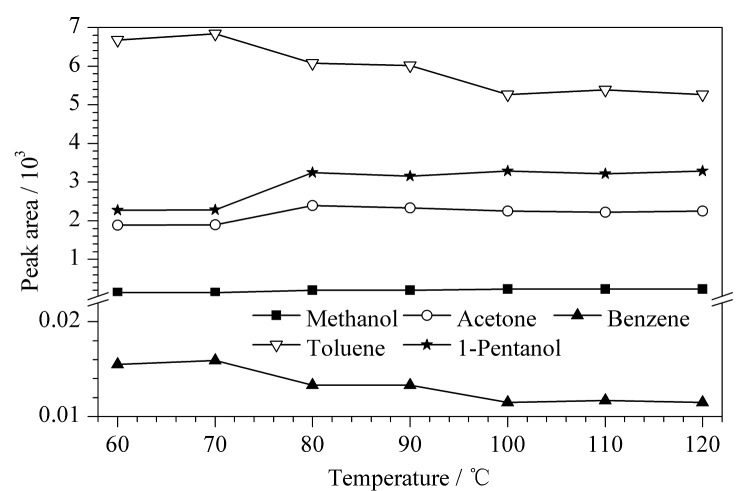
平衡温度对供试品中5种溶剂峰面积的影响

从图1可以看出,在60~80 ℃的范围内,随着顶空平衡温度的升高,甲醇、丙酮、1-戊醇3种溶剂的峰面积随着平衡温度的升高而增大。苯和甲苯的峰面积随着平衡温度升高呈现降低的趋势,变化比较缓慢。随着温度继续升高,5种溶剂峰面积的变化趋势并不明显。考虑到继续升高温度会增加顶空瓶漏气及爆裂的危险,同时会使杂质和水分进入色谱柱而干扰目标物的测定,最终选择顶空平衡温度为80 ℃。

#### 2.1.2 顶空平衡时间

平衡时间是各种残留溶剂挥发到顶空气相中并达到气液平衡所需的时间,其本质取决于被测组分分子的扩散速度。平衡时间短,溶剂挥发不完全。平衡时间过长,不仅会增加检测时间,还可能影响顶空瓶的密封性,进而导致检测效率下降^[19]^。分别取供试品溶液在10、20、30、45 min 4个平衡时间条件下进行GC测定,结果如图2。综合考虑,选择顶空平衡时间为20 min。

**图2:**
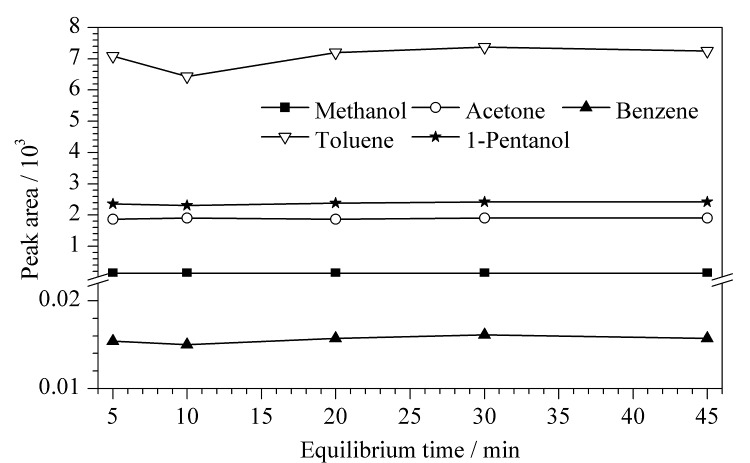
平衡时间对供试品中5种溶剂峰面积的影响

### 2.2 方法学考察

#### 2.2.1 系统适用性试验

以混合标准溶液为测试液,重复测定6次,分别计算5种溶剂峰面积的RSD,结果见图3。结果表明:混合对照品溶液中各溶剂的色谱峰面积的RSD为0.64%~1.84%,均小于2.0%。各溶剂峰的理论塔板数均大于10000,相邻溶剂峰之间的分离度均大于2.0,检测方法满足系统适用性要求。

**图3:**
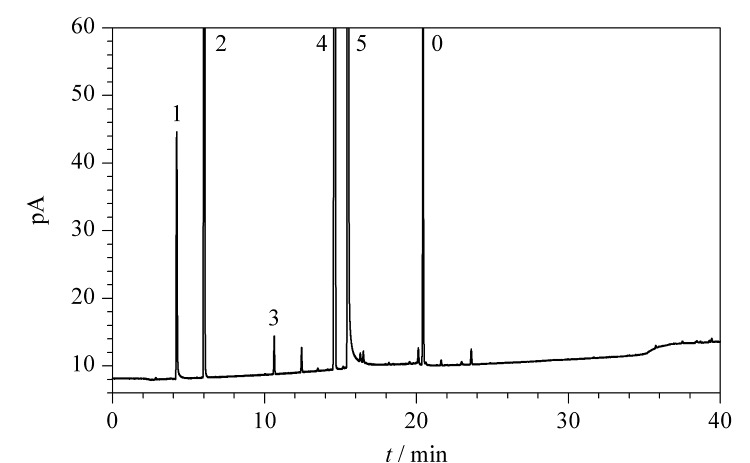
系统适用性试验的气相色谱图

#### 2.2.2 线性关系

分别取各系列混合标准溶液,在优化条件下进行HS-GC检测,以5种溶剂的峰面积(*y*)为纵坐标,质量浓度(*x*)为横坐标,绘制标准曲线,各溶剂的线性方程如表1。结果表明:5种残留溶剂在选择的浓度范围内线性关系良好,相关系数*r*值均大于0.990。

**表1:** 5种溶剂的线性范围、回归方程、相关系数、检出限与定量限

Solvent	Linear range/(μg/mL)		Regression equation	r	LOD/(ng/mL)		LOQ/(ng/mL)	
Methanol	72-	216	y=0.7211x+0.1107	0.9980		2.88		11.5
Acetone	120-	360	y=6.126x+71.4	0.9993		0.011		0.043
Benzene	0.048-	0.144	y=146.09x-1.2225	0.9983		0.90		3.6
Toluene	21.36-	64.08	y=146.25x-483.08	0.9982		0.24		0.96
1-Pentanol	120-	360	y=7.3536x+97.755	0.9988		0.024		0.096

*y*: peak area of solvent; *x*: mass concentration, μg/mL.

#### 2.2.3 检出限与定量限

采用逐级稀释法,以*S/N*≥3确定方法的检出限(LOD),以*S/N*≥10确定方法的定量限(LOQ),结果如表1。结果表明,方法灵敏度满足样品定量分析及《中国药典》的限度要求。

#### 2.2.4 准确度试验

取伊班膦酸钠原料药约600 mg共9份,精密称定,置于10 mL量瓶中,分别精密加入高、中、低3种不同质量浓度的混合标准溶液并稀释至刻度,摇匀,每种浓度平行制备3份。再精密量取上述溶液各5 mL,进行HS-GC检测,计算5种溶剂的回收率,结果见表2。

**表2:** 伊班膦酸钠中5种溶剂在3个水平下的回收率和RSD(*n*=3)

Solvent	Added/ (mg/g)	Found/ (mg/g)	Recovery/ %	RSD/ %
Methanol	2.400	2.444	101.9	2.49
	3.000	3.010	100.3	1.29
	3.600	3.574	99.3	1.57
Acetone	4.000	3.995	99.9	1.72
	5.000	4.956	99.1	0.63
	6.000	5.912	98.5	1.04
Benzene	0.0016	0.00138	86.3	1.65
	0.0020	0.00190	94.8	1.93
	0.0024	0.00232	96.9	1.55
Toluene	0.712	0.6255	87.9	1.63
	0.890	0.8429	94.7	1.76
	1.068	0.9780	91.6	1.40
1-Pentanol	4.000	4.034	100.9	2.26
	5.000	4.928	98.6	0.73
	6.000	5.912	98.5	1.31

由表2可以看出,各溶剂的平均回收率为86.3%~101.9%, RSD为0.63%~2.49%,满足平均回收率在90%~110%范围内、RSD(*n*=3)不大于5.0%的要求,说明本方法准确度良好。

#### 2.2.5 重复性试验

按1.2.3节的步骤平行制备6份供试品溶液,分别精密量取5 mL,进行HS-GC测定,根据标准曲线计算各溶剂的含量。试验结果表明,各样品中各溶剂峰的含量相差较小,甲醇、丙酮、苯、甲苯、1-戊醇含量的平均值分别为0.33%、0.51%、0.0002%、0.08%和0.49%, RSD为0.63%~1.84%,说明该方法的重复性较好。

#### 2.2.6 耐用性

分别改变试验参数,采用载气流速0.9、1.0、1.1 mL/min,顶空平衡温度90、100 ℃,顶空平衡时间20、30 min,考察试验参数小幅度改变对供试品溶液中溶剂含量的影响。试验结果表明,流速、顶空平衡温度和平衡时间的微小变化对供试品溶液中各种溶剂的含量几乎没有影响,说明方法耐用性良好。

### 2.3 与已有报道方法的比较

与现有文献相比(见表3),本方法的检出限和定量限远低于文献报道,说明本研究建立的方法灵敏度和特异性更好。

**表3:** 本研究方法与文献报道方法的对比

Ref.	Injection mode	Solvent	LOD/ (ng/mL)	LOQ/ (ng/mL)
This study	headspace	methanol	2.88	11.5
		acetone	0.011	0.043
		benzene	0.90	3.6
		toluene	0.24	0.96
		1-pentanol	0.024	0.096
[15]	direct	methanol	500	unreported
		acetone	200	
		ethanol	200	
		chlorobenzene	100	
[16]	direct	methanol	250	750
		acetone	210	630
		ethanol	80	250
		toluene	600	1810
[20]	headspace	toluene	350	unreported
		chlorobenzene	1130	

### 2.4 实际样品的快速检测

取6批供试品,平行进样3针,在优化的HS-GC色谱条件下测定溶剂残留,计算各残留溶剂的含量。结果表明,在6批检测样品中,均未检出5种残留溶剂,但有限的样品数量不能代表全部,为了药品的安全及稳定性,对伊班膦酸钠原料药的残留溶剂的监测仍十分必要。

## 3 结论

本研究成功地建立了一种简便、高效的残留溶剂测定方法。这一方法能够可靠地对伊班膦酸钠原料药中的5种溶剂残留进行精确的定量分析。本方法提供了有效的手段来监测和控制伊班膦酸钠原料药的质量,填补了《中国药典》中伊班膦酸钠质量标准的空白,有助于提高伊班膦酸钠产品的质量和安全性,从而对医药行业产生积极的影响。
